# Supplementary material for: The rice NLR pair Pikp-1/Pikp-2 initiates cell death through receptor cooperation rather than negative regulation
Source: PLoS One. 2020 Sep 15;15(9):e0238616. doi: 10.1371/journal.pone.0238616 (PMC7491719; doi:10.1371/journal.pone.0238616)
Supplement: S1 Raw images — (PDF) [file pone.0238616.s002.pdf]

Fig 4A)

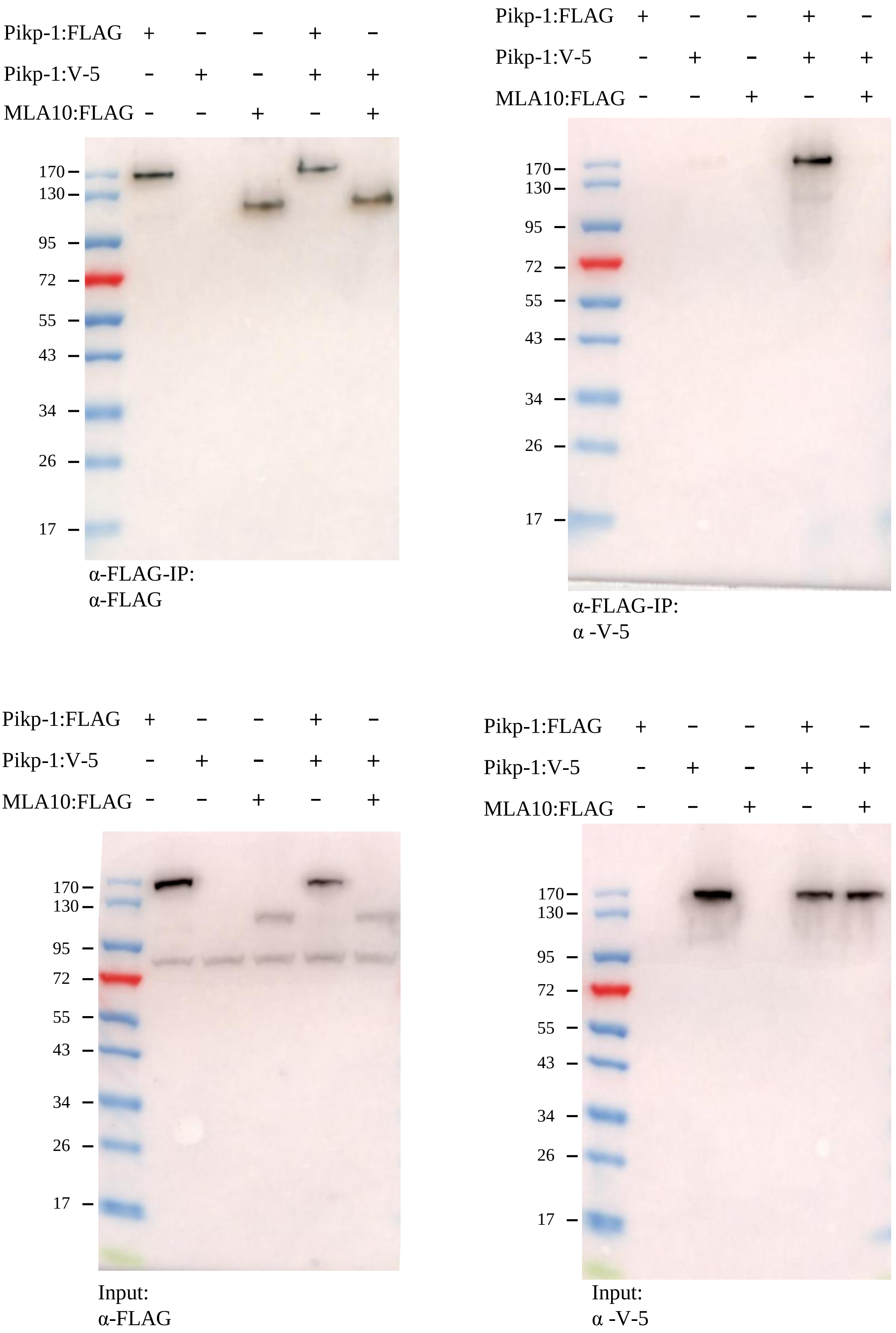

Pikp-1:FLAG + − − + −
Pikp-1:V-5 - + − + +
MLA10:FLAG - − + − +
170
130
95
72
55
43
34
26
17
α-FLAG-IP:
α-FLAG
Pikp-1:FLAG + − − + −
Pikp-1:V-5 - + − + +
MLA10:FLAG - − + − +
170
130
95
72
55
43
34
26
17
α-FLAG-IP:
α -V-5
Pikp-1:FLAG + − − + −
Pikp-1:V-5 - + − + +
MLA10:FLAG - − + − +
170
130
95
72
55
43
34
26
17
Input:
α-FLAG
Pikp-1:FLAG + − − + −
Pikp-1:V-5 - + − + +
MLA10:FLAG - − + − +
170
130
95
72
55
43
34
26
17
Input:
α -V-5

Fig 4B)

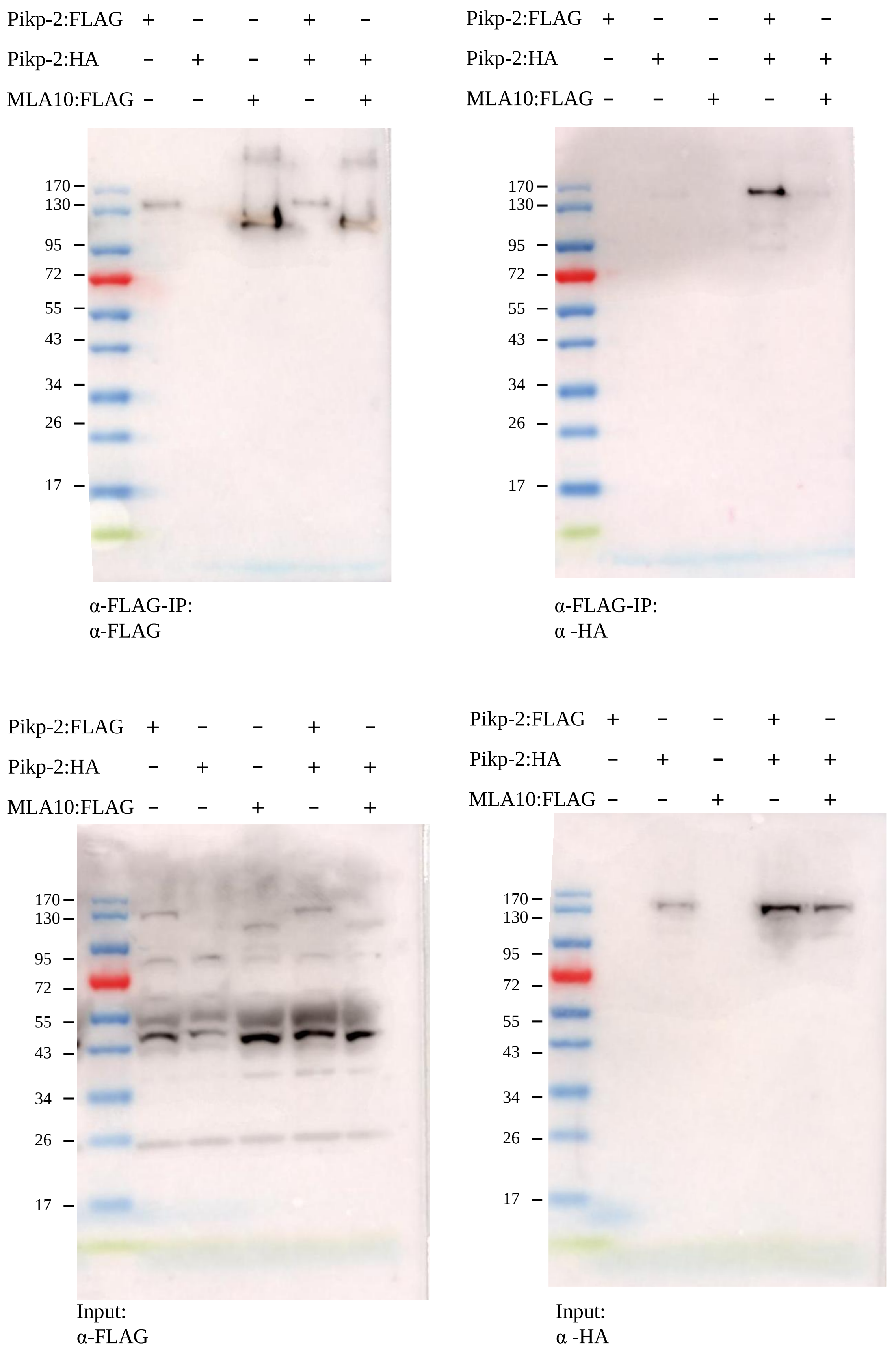
Pikp-2:FLAG + – – + –
Pikp-2:HA – + – + +
MLA10:FLAG – – + – +
170
130
95
72
55
43
34
26
17
α-FLAG-IP:
α-FLAG
Pikp-2:FLAG + – – + –
Pikp-2:HA – + – + +
MLA10:FLAG – – + – +
170
130
95
72
55
43
34
26
17
α-FLAG-IP:
α -HA
Pikp-2:FLAG + – – + –
Pikp-2:HA – + – + +
MLA10:FLAG – – + – +
170
130
95
72
55
43
34
26
17
Input:
α-FLAG
Pikp-2:FLAG + – – + –
Pikp-2:HA – + – + +
MLA10:FLAG – – + – +
170
130
95
72
55
43
34
26
17
Input:
α -HA

Fig 5A)

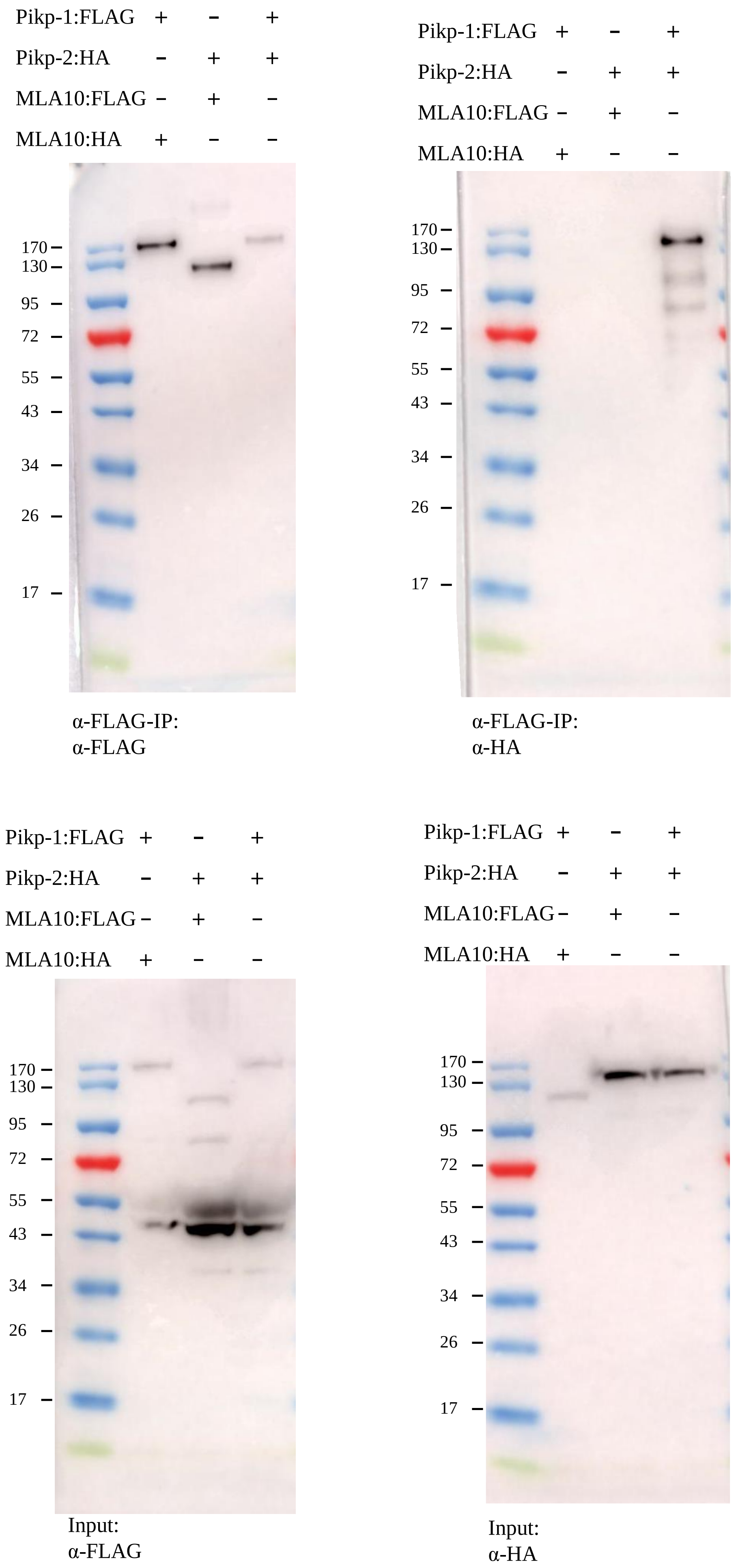

Pikp-1:FLAG + − +
Pikp-2:HA − + +
MLA10:FLAG − + −
MLA10:HA + − −
170
130
95
72
55
43
34
26
17
α-FLAG-IP:
α-FLAG
α-FLAG-IP:
α-HA
Input:
α-FLAG
Input:
α-HA

Fig 5B)

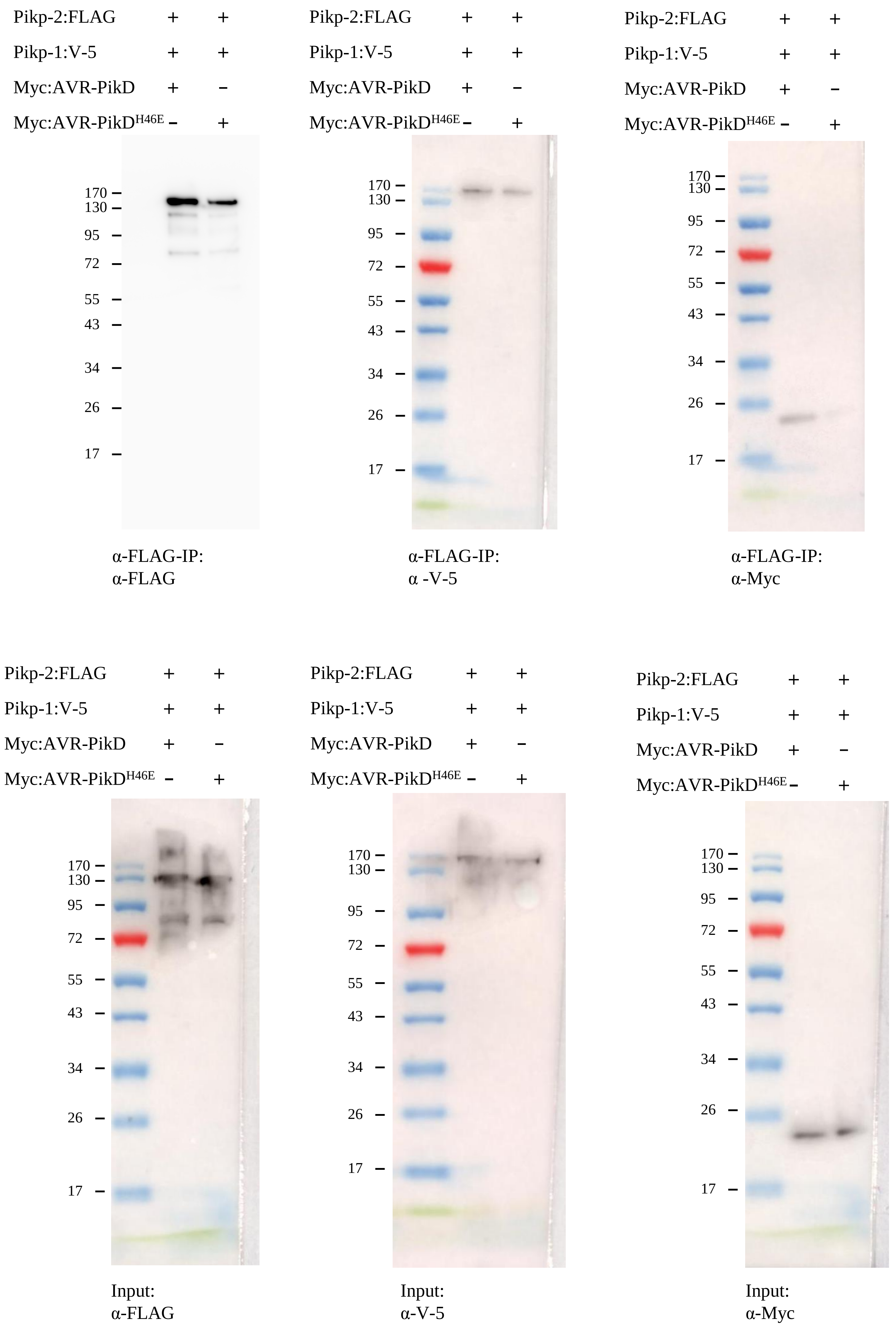

Pikp-2:FLAG + +
Pikp-1:V-5 + +
Myc:AVR-PikD + –
Myc:AVR-PikD^{H46E} – +
170
130
95
72
55
43
34
26
17
α-FLAG-IP:
α-FLAG
α-FLAG-IP:
α -V-5
α-FLAG-IP:
α-Myc
Input:
α-FLAG
Input:
α-V-5
Input:
α-Myc

Fig S2

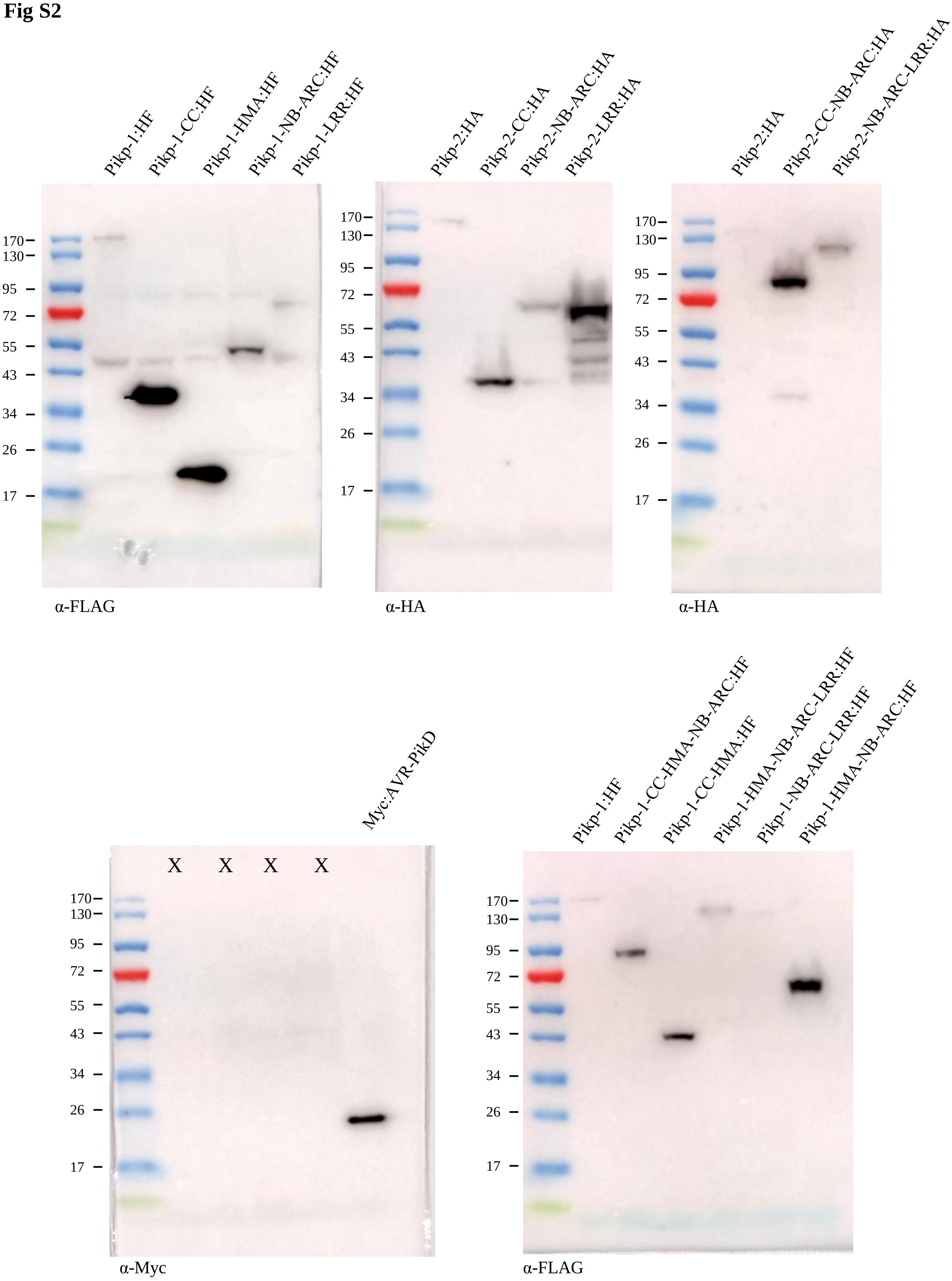
Pikp-1:HF
Pikp-1-CC:HF
Pikp-1-HMA:HF
Pikp-1-NB-ARC:HF
Pikp-1-LRR:HF
170
130
95
72
55
43
34
26
17
α-FLAG
Pikp-2:HA
Pikp-2-CC:HA
Pikp-2-NB-ARC:HA
Pikp-2-LRR:HA
170
130
95
72
55
43
34
26
17
α-HA
Pikp-2:HA
Pikp-2-CC-NB-ARC:HA
Pikp-2-NB-ARC-LRR:HA
170
130
95
72
55
43
34
26
17
α-HA
Myc:AVR-PikD
X X X X
170
130
95
72
55
43
34
26
17
α-Myc
Pikp-1:HF
Pikp-1-CC-HMA-NB-ARC:HF
Pikp-1-CC-HMA:HF
Pikp-1-HMA-NB-ARC-LRR:HF
Pikp-1-NB-ARC-LRR:HF
Pikp-1-HMA-NB-ARC:HF
170
130
95
72
55
43
34
26
17
α-FLAG

Fig S3B

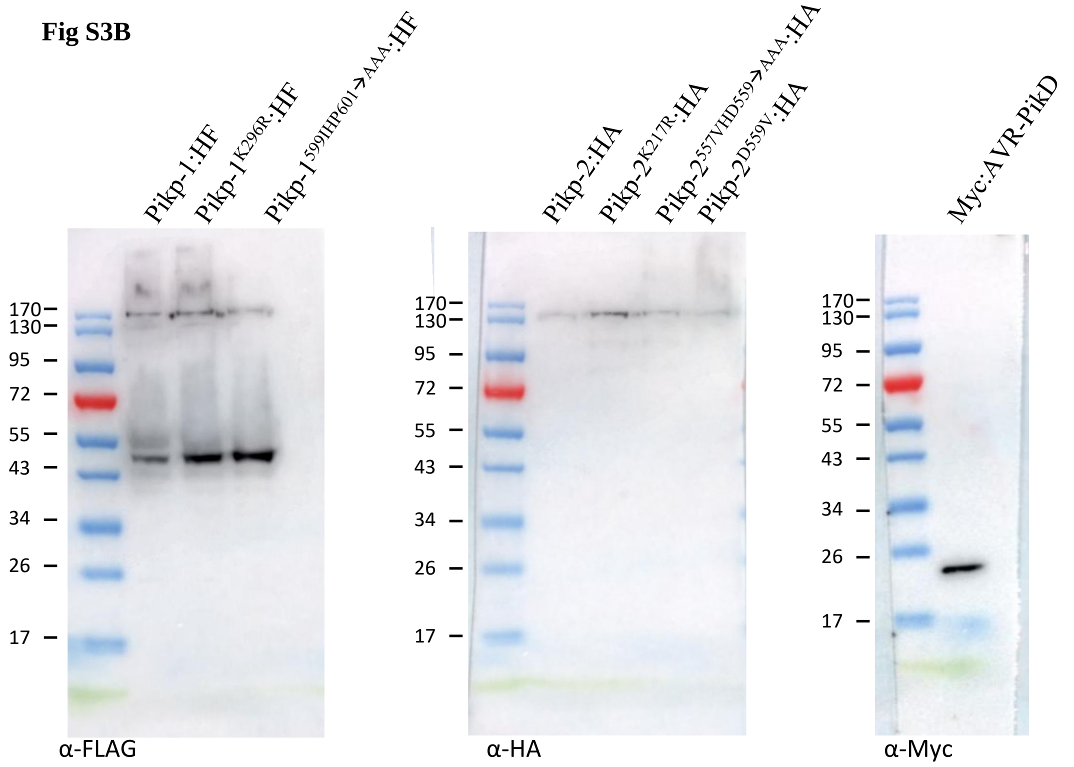

Pikp-1:HF
Pikp-1K296R:HF
Pikp-1599IHP601→AAA:HF
Pikp-2:HA
Pikp-2K217R:HA
Pikp-2557VHD559→AAA:HA
Pikp-2D559V:HA
Myc:AVR-PikD
170
130
95
72
55
43
34
26
17
α-FLAG
α-HA
α-Myc

Fig S4A)

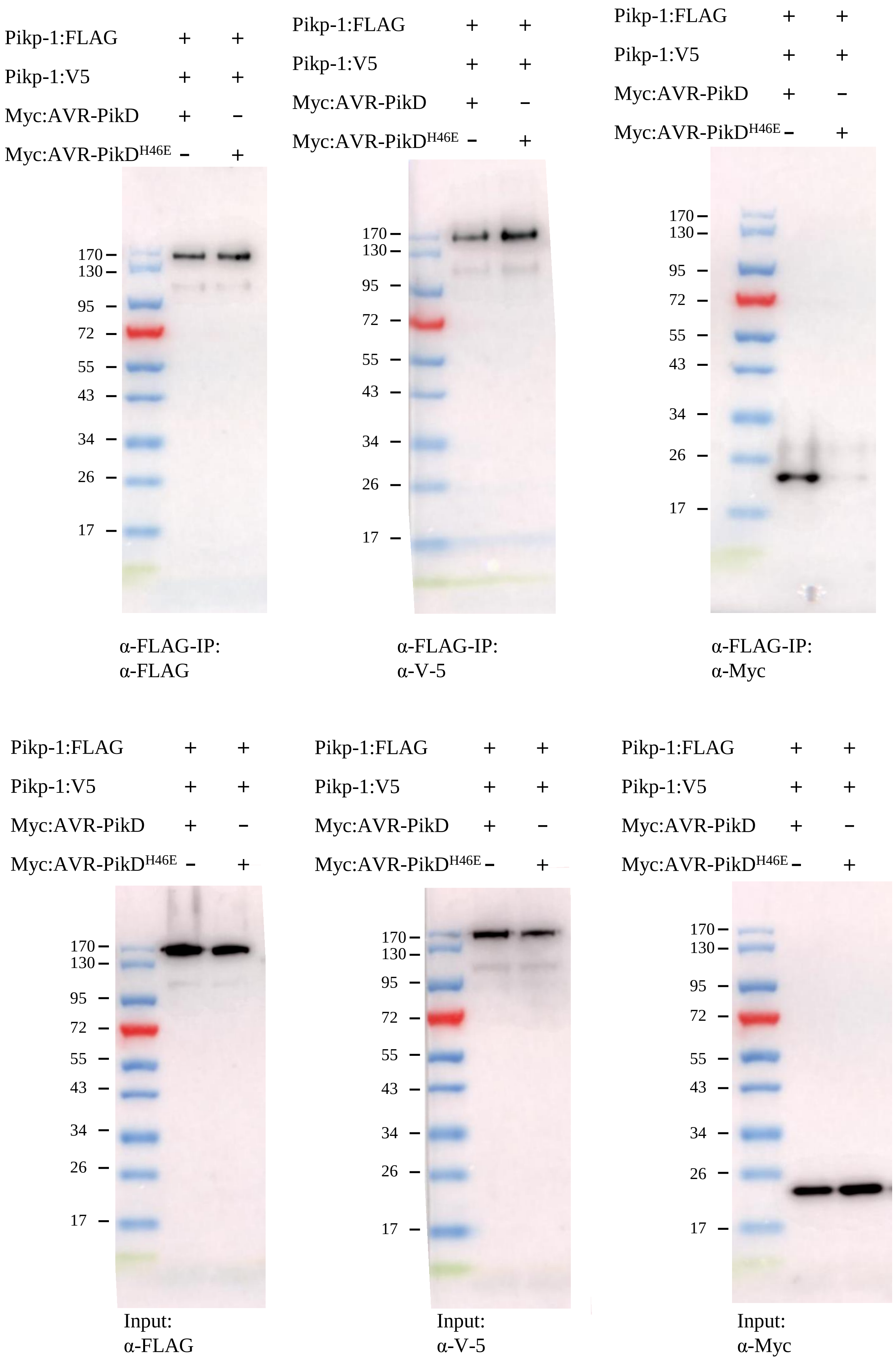

Pikp-1:FLAG + +
Pikp-1:V5 + +
Myc:AVR-PikD + –
Myc:AVR-PikD^{H46E} – +
170
130
95
72
55
43
34
26
17
α-FLAG-IP:
α-FLAG
Pikp-1:FLAG + +
Pikp-1:V5 + +
Myc:AVR-PikD + –
Myc:AVR-PikD^{H46E} – +
170
130
95
72
55
43
34
26
17
α-FLAG-IP:
α-V-5
Pikp-1:FLAG + +
Pikp-1:V5 + +
Myc:AVR-PikD + –
Myc:AVR-PikD^{H46E} – +
170
130
95
72
55
43
34
26
17
α-FLAG-IP:
α-Myc
Pikp-1:FLAG + +
Pikp-1:V5 + +
Myc:AVR-PikD + –
Myc:AVR-PikD^{H46E} – +
170
130
95
72
55
43
34
26
17
Input:
α-FLAG
Pikp-1:FLAG + +
Pikp-1:V5 + +
Myc:AVR-PikD + –
Myc:AVR-PikD^{H46E} – +
170
130
95
72
55
43
34
26
17
Input:
α-V-5
Pikp-1:FLAG + +
Pikp-1:V5 + +
Myc:AVR-PikD + –
Myc:AVR-PikD^{H46E} – +
170
130
95
72
55
43
34
26
17
Input:
α-Myc

Fig S4B)

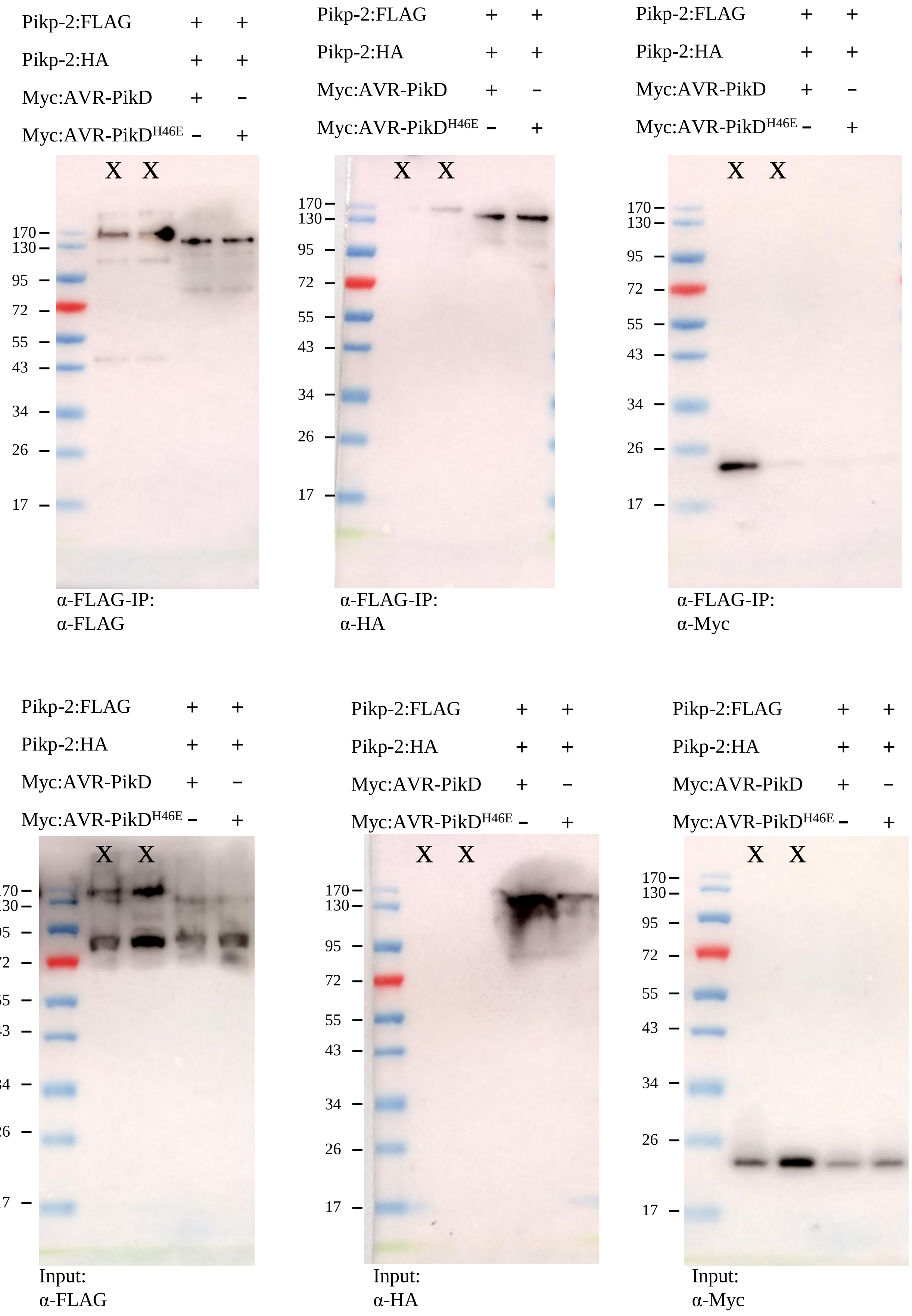
Pikp-2:FLAG + +
Pikp-2:HA + +
Myc:AVR-PikD + –
Myc:AVR-PikDH46E – +
X X
170
130
95
72
55
43
34
26
17
α-FLAG-IP:
α-FLAG
α-FLAG-IP:
α-HA
α-FLAG-IP:
α-Myc
Input:
α-FLAG
Input:
α-HA
Input:
α-Myc
